# Effect of a new nursing organization management mode constructed by appreciative inquiry on gynecological tumor patients in the perioperative period

**DOI:** 10.1590/1980-220X-REEUSP-2025-0016en

**Published:** 2025-10-27

**Authors:** Chuntao Fang, Jing Yang

**Affiliations:** 1 Huai’an Second People’s Hospital, Department of Gynecology, Huai’an, China.

**Keywords:** Surveys and Questionnaires, Gynecology, Neoplasms, Nursing, Health Management, Perioperative Period, Inquéritos e Questionários, Ginecologia, Neoplasias, Enfermagem, Gestão em Saúde, Período Perioperatório

## Abstract

**Objective:**

To evaluate the effect of a new nursing organizational management model based on appreciative inquiry on gynecological tumor patients during the perioperative period.

**Method:**

A retrospective analysis was conducted on 204 gynecological tumor patients who underwent surgical treatment between June 2021 and June 2024. Patients were assigned to a control group (n = 102; routine nursing organization management mode) or a study group (n = 102; new nursing organization management mode constructed by appreciative inquiry) according to their nursing mode during the perioperative period.

**Results:**

Both groups had increased World Health Organization Quality of Life Scale Brief Version (WHOQOL-BREF) scores and decreased Pelvic Floor Distress Inventory-Short Form 20 (PFDI-20) scores compared to before the intervention. The study group had higher WHOQOL-BREF scores and lower PFDI-20 scores than the control group (p < 0.05). Scores on the Social Impact Scale in each dimension and the total score declined in both groups compared to baseline, with lower scores observed in the study group than in the control group (p < 0.05).

**Conclusion:**

The new nursing organizational management model developed using appreciative inquiry can optimize perioperative indicators, improve pelvic floor dysfunction, and enhance psychological resilience.

## RESUMO

**Objetivo:**

Avaliar o efeito de um novo modelo de gestão de organizações de enfermagem, construído por meio de uma investigação apreciativa, em pacientes com tumores ginecológicos no período perioperatório.

**Método:**

Foi realizada uma análise retrospectiva em 204 pacientes com tumor ginecológico submetidas a tratamento cirúrgico entre junho de 2021 e junho de 2024. As pacientes foram distribuídas em um grupo controle (n = 102, um modelo de gestão de organização de enfermagem de rotina) e um grupo de estudo (n = 102, um novo modelo de gestão de organização de enfermagem construído por meio de investigação apreciativa) de acordo com os diferentes modelos de enfermagem no período perioperatório.

**Resultados:**

As pontuações da Escala de Qualidade de Vida da Organização Mundial da Saúde Versão Resumida (WHOQOL-BREF) aumentaram, enquanto as pontuações do Inventário de Distúrbios do Assoalho Pélvico Forma Resumida 20 (PFDI-20) diminuíram em ambos os grupos em comparação com as pontuações anteriores à intervenção. O grupo de estudo apresentou pontuações WHOQOL-BREF mais altas e pontuações PFDI-20 mais baixas do que as do grupo de controlo (p < 0,05). A pontuação da Escala de Impacto Social em cada dimensão e a pontuação total diminuíram em ambos os grupos em comparação com os valores anteriores à intervenção, e foram mais baixas no grupo de estudo do que no grupo de controlo (p < 0,05).

**Conclusão:**

O novo modo de gestão da organização de enfermagem construído pela investigação apreciativa pode otimizar os indicadores perioperatórios, melhorar a disfunção do pavimento pélvico e aumentar a resiliência psicológica.

## INTRODUCTION

Ovarian cancer (OC), cervical cancer (CCA), endometrial cancer (EC), and uterine fibroids (UM) are common tumors of the female reproductive system. Patients often present with pelvic compression, vaginal bleeding, and, in severe cases, pelvic floor dysfunction (PFD), which has a serious adverse impact on the psychological, physiological, and social functions and quality of life (QoL) of patients^(1,2)^. Currently, surgery is an important treatment for gynecological tumors. However, since the uterus and ovaries are vital organs for women, and surgery is invasive, most patients may experience fear, anxiety, and other negative emotions due to concerns about surgical trauma, postoperative recovery, and fertility. This increases the risk of intraoperative cardiovascular stress responses and hinders the successful implementation of surgery and postoperative recovery^(3,4)^. Therefore, active and effective nursing measures taken during the perioperative period are very important for stabilizing patients’ emotions and helping them develop positive coping strategies.

Appreciative inquiry is an effective approach to promoting organizational change. It does so by systematically discovering and activating the maximum efficiency and capability of organizational life^(5, 6)^. Appreciative inquiry plays an important role in improving human resource management, enhancing team capacity building, developing clinical nursing practice, and promoting organizational change in nursing. In a qualitative study of intensive care unit interns during the pandemic, appreciative inquiry prompted the interns to actively consider and redesign their workflow, work support, and personal development, ultimately helping them achieve organizational goals^(7)^. The appreciative inquiry concept has also been applied to organizational change in primary oral healthcare, using a 4D (discovery, dream, design, destiny) cycle model throughout the process^(8)^. By improving organizational management and increasing resources, the desire of organizational members to integrate oral health into primary healthcare was strengthened, successfully implementing the organizational strategic plan. Appreciative inquiry has been introduced to assist Canadian family physicians in providing the best support for patients in need of palliative care in community medical homes^(9)^. Appreciative inquiry was more effective than other management methods at strengthening communication and cultivating teamwork among caregivers, such as family physicians, and at helping establish good nurse-patient relationships, thereby improving the quality of palliative care.

In this study, a new nursing organizational management model based on appreciative inquiry was applied to the nursing intervention for gynecological tumor patients undergoing surgery, and its effects on perioperative indicators, psychological state, quality of life (QoL), and nursing satisfaction were assessed.

## METHOD

### Study Design and Subjects

In this retrospective study, the required sample size was determined based on the equation for comparing two proportions: n = 2 × (Z_α/2_ + Z_β_)^2^ × p(1 – p)/d^2^ where n = sample size per group, Z_α/2_ = Z-value corresponding to the desired significance level (for a = 0.05, two-tailed, Z_α/2_ = 1.96), Z_β_ = Z-value corresponding to power (for power = 80%, Z_β_ = 0.84), P is the estimated incidence rate in the control group, based on previous studies or pilot data and assumed to be 50%. D is the anticipated clinically significant difference between the groups, set as a 20% improvement. Using this equation, n was calculated to be 98. Considering an expected dropout rate of approximately 5%, the final sample size was adjusted to 102 participants per group for a total of 204 participants.

From June 2021 to June 2024, data were collected from 204 gynecological tumor patients undergoing surgical treatment in our hospital. According to different nursing modes in the perioperative period, patients were assigned to a control group (n = 102; routine nursing organization management mode) or a study group (n = 102; new nursing organization management mode constructed by appreciative inquiry).

### Ethical Aspects Aubsection

The Research Ethics Committee of Huai’an Second People’s Hospital approved this study (No. JSHASH2021105).

### Inclusion and Exclusion Criteria

The inclusion criteria were as follows: 1) Patients who met the diagnostic criteria for OC, CCA, EC, or UM^(10, 11, 12, 13)^ and who were confirmed by:

- Hysteroscopy (26105FA, KARL STORZ, Germany)
- Color Doppler ultrasound diagnostic instrument (IU22, Philips, the Netherlands)
- Colposcopy (DKY-1611, Dalian Care Medical Equipment Co., Ltd.), and pathological biopsy; 2) patients with complete clinical data; 3) patients undergoing surgical treatment for the first time; and 4) patients without psychiatric disorders (e.g., schizophrenia or bipolar disorder).

The exclusion criteria included: 1) patients unable to cooperate in completing the scale due to cognitive or communication disorders; 2) patients who developed other serious diseases during the intervention; 3) patients complicated by other neoplastic diseases; 4) patients with an estimated survival time of less than six months; 5) patients complicated by distant organ metastases (e.g., lung, liver, brain); and 6) patients aged less than 20 years.), 7) severe liver, kidney, heart, or lung dysfunction, 8) severe impairment of limb function or other diseases that seriously affect limb movement (e.g., osteoarthritis), 9) age <20 years, and 10) hematological, communicable, or infectious disease.

### Management Mode for Control Group

A routine nursing organizational management model was adopted. Specifically, health brochures detailing the causes, hazards, surgical protocols, and postoperative precautions of gynecological tumors were distributed to patients upon admission. The brochures also instructed patients to relax and accept surgery. Seven to ten days after surgery, patients were guided and assisted in routine pelvic floor muscle rehabilitation training. This involved consciously contracting the vaginal, urethral, and anal muscles after emptying the bladder for five to ten seconds, followed by ten seconds of relaxation. Patients performed this exercise ten to fifteen minutes per session, three times per day. The intervention lasted four weeks.

### Management Mode for Study Group

We adopted a new nursing organization management mode constructed by appreciative inquiry. Our institution introduced this model through a structured implementation process. First, nursing managers and core staff received targeted training. Then, a dedicated nursing quality improvement team was formed. The team then led department-wide workshops and discussions to identify strengths, establish shared goals, and redesign perioperative nursing processes collaboratively. Standardized protocols and communication models based on appreciative inquiry principles were developed and incorporated into daily practice.

1) Discovery: Experienced medical staff participated in professional training in the appreciative inquiry method to understand the 4D (discovery, dream, design, destiny) cycle. A relaxed and harmonious meeting atmosphere was created, and all medical staff were positively and optimistically encouraged to talk about impressive cases and their positive impacts on their work, sharing high-quality nursing experiences.

2) Dream: Based on sharing the best nursing experiences, the nursing staff was encouraged to imagine what nursing work might be like in the next five to ten years, combining their own advantages and potential. They were also encouraged to boldly redesign personal role allocation, departmental organizational structure, and nursing work processes. Team members discussed ways to achieve organizational goals and helped each other turn their ideas into specific, result-oriented measures.

3) Design: Through one-on-one in-depth interviews combined with the best working conditions, personal potential, and organizational advantages, the nursing staff was encouraged to consider how to promote the realization of the future vision. Team members jointly formulated a series of effective and feasible nursing protocols, specifically: a) PFM training: Based on routine PFM training, urine suppression training and biofeedback training were provided from the fifth day postoperatively, according to the patients’ conditions. These training sessions were assisted by the PFM electric biofeedback instrument (Huayu Medical Technology [Wuhan] Co., Ltd., HD-V8, EHXB 20190157). The content of the training, specific operation methods, and relevant precautions were explained in detail, taking into account the patients’ educational backgrounds. b) Continuous nursing: Nurse-patient WeChat groups were established, and WeChat Official Accounts were created to regularly send messages about postoperative precautions, rehabilitation training videos, and postoperative nursing measures for gynecological tumors. Family members were encouraged to care for and supervise patients in their rehabilitation training. Special lectures on exercise were held regularly, and family members were invited to attend. During these lectures, rehabilitation training such as going up and down stairs, bodybuilding exercises, and abdominal breathing were introduced. Patients with good postoperative recovery were invited to share their experiences. c) Psychological Nursing: Health education activities with the theme “I Know You Know” were carried out three days preoperatively. Patients were encouraged to introduce themselves and communicate with each other. The relationship between postoperative rehabilitation and emotional management was explained to gynecological tumor patients through videos and PowerPoint presentations. A second activity, “Travel with Trust and Unobstructed Communication,” was carried out two days before surgery to encourage patients to communicate, experience different communication modes, and promote positive information flow. A patient-to-patient exchange meeting with the theme of “Self and Vision Reconstruction” was held one day before surgery to encourage patients to imagine better lives after surgery and share their ideas with others. d) Imaginative relaxation training: Patients were guided through relaxation training using pictures of the seaside, beach, and other scenes, as well as background music. For example, patients were asked to imagine a seaside scene with pictures of sunsets and coconut trees, while music with the sounds of ocean waves and swaying coconut trees played in the background. Guiding words were also provided: “You are walking along the seaside with good friends, enjoying the sunset and the clouds. The breeze is blowing, and everything is beautiful...” This was done once before bed at night or before an afternoon nap.

4) Destiny: The best nursing experiences and achievements were shared to help nursing staff build hope around deep-level nursing goals and career visions. This helped them maintain positive motivation for continuous learning and adjustment, as well as active participation in nurse-patient scene exercises to improve communication skills. The intervention lasted four weeks.

To ensure consistency in applying the appreciative inquiry–based nursing organization management model over the three-year study period, a standardized training manual and implementation protocol were developed and implemented. All newly hired nurses in the study group received structured onboarding that included appreciative inquiry theory, the 4D cycle process, and practical applications tailored to perioperative care. Additionally, monthly quality review meetings and refresher training sessions were conducted to maintain fidelity to the appreciative inquiry approach and ensure the core principles were upheld despite staff turnover.

### Evaluation of Outcomes

1. The following indicators were evaluated before and after the four-week intervention:
  - Psychological resilience: Psychological resilience was assessed using the 25-item Connor-Davidson Resilience Scale (CD-RISC), which covers five dimensions: personal competence/tenacity (eight items)^(14)^, positive acceptance of change (five items), tolerance of negative affect (seven items), spiritual influences (two items), and self-control (three items). Items are scored on a 5-point Likert scale: never (0), rarely (1), sometimes (2), often (3), and always (4). The possible scores range from 0 to 100, with higher scores indicating greater resilience.
  - Posttraumatic Growth Level: Posttraumatic positive thinking attitudes were assessed using the 21-item Posttraumatic Growth Inventory (PTGI), which consists of five dimensions^(15)^: spiritual change (two items), relating to others (seven items), new possibilities (five items), appreciation of life (three items), and personal strength (four items). Items are scored on a six-point Likert scale ranging from 0 (never) to 5 (maximal). Scores range from 21 to 126, with higher scores indicating stronger positive psychological change and growth.
  - QoL: The QoL of the two groups was evaluated using the World Health Organization Quality of Life Scale Brief Version (WHOQOL-BREF), which includes 26 items across four dimensions: social relationships (three items)^(16)^, environment (eight items), psychology (six items), and physical health (seven items). Items are scored on a 5-point Likert scale (1–5 points), and some items are scored in reverse. Scores range from 26 to 130 points; a lower score indicates a worse QoL.
  - Severity of Pelvic Floor Distress (PFD): The Pelvic Floor Distress Inventory-Short Form 20 (PFDI-20) was used to evaluate PFD severity in the two groups^(17)^. The scale consists of 20 items, each scored on a 4-point Likert scale: no impact (1), mild impact (2), moderate impact (3), and severe impact (4). Scores range from 20 to 80, with lower scores indicating milder PFD.
  - Stigma: The stigma experienced by the two groups was assessed using the 24-item Social Impact Scale (SIS), which includes four dimensions^(18)^: internalized shame (five items, 5–20 points); social isolation (seven items, 7–28 points); financial insecurity (three items, 3–12 points); and social rejection (nine items, 9–36 points). Each item is scored on a four-point scale, with a total score ranging from 24 to 96 points. A higher score indicates greater stigma.
2. Perioperative Indicators and Nursing Satisfaction: Postoperative exhaust time and length of stay were recorded for the two groups. A hospital-designed questionnaire was used to evaluate nursing satisfaction and covered four dimensions: nursing attitude, nursing level, health education, and nursing management. The total possible score was 100 points, and higher scores indicated higher satisfaction. To ensure the quality of the questionnaire, a preliminary evaluation of its reliability and validity was conducted. Internal consistency reliability was assessed using Cronbach’s alpha, which was found to be 0.86, indicating good reliability. Content validity was evaluated by a panel of five senior nursing experts, yielding a content validity index of 0.89. These results supported the questionnaire’s reliability and validity for use in this study.

### Statistical Analysis

The SPSS 23.0 software was used for the analysis. The measurement data were described using the mean ± standard deviation (x ± s) and were compared using an independent- samples t-test between two groups and a paired-samples t-test within the group. Count data were described as a percentage [n (%)] and underwent a chi-squared test. Multivariate analyses or subgroup/interaction analyses were not performed, which may limit control of potential confounding factors. Missing data were minimal (less than 5%) and were handled using complete-case analysis. A two-sided p-value <0.05 was considered statistically significant.

## RESULTS

### Baseline Clinical Data

There were no significant differences in the educational level, age, course of disease, tumor type, body mass index, surgical method, or marital status between the two groups (p > 0.05) (Table 1).

**Table 1:** General data of two groups – Huai’an, Jiangsu Province, China, 2021.

Indicator		Study group (n = 102)	Control group (n = 102)	Statistical value	p
Educational level [n (%)]	Senior high school and above	58 (56.86)	53 (51.96)	χ^2^ = 0.494	0.482
	Junior high school and below	44 (43.14)	49 (48.04)		
Age (x̄ ± s, year)		52.57 ± 6.13	53.04 ± 6.18	t = 0.545	0.586
Course of disease (x̄ ± s, month)		10.66 ± 0.98	10.74 ± 1.05	t = 0.563	0.574
Tumor type [n (%)]	OC	7 (6.86)	6 (5.88)	χ^2^ = 1.203	0.752
	CCA	8 (7.84)	7 (6.86)		
	EC	10 (9.80)	15 (14.71)		
	UM	77 (75.49)	74 (72.55)		
Body mass index (x ± s, kg/m^2^)		22.14 ± 0.85	22.25 ± 0.88	t = 0.908	0.365
Surgical mode [n (%)]	Radical surgery	51 (50.00)	46 (45.10)	χ^2^ = 0.498	0.780
	Uterus-preserving surgery	25 (24.51)	27 (26.47)		
	Total hysterectomy	26 (25.49)	29 (28.43)		
Marital status [n (%)]	Married	87 (85.29)	83 (81.37)	χ^2^ = 1.272	0.529
	Unmarried	9 (8.82)	14 (13.73)	χ^2^ = 0.498	0.780
	Divorced/widowed/separated	51 (50.00)	46 (45.10)		

### Psychological Resilience and Posttraumatic Growth Level

After four weeks of the intervention, both groups had higher scores on the CD-RISC and PTGI than before the intervention. The study group had higher scores than the control group (p < 0.05) (Table 2).

**Table 2:** CD-RISC and PTGI scores before and after four weeks of intervention (*x̄* ± s, point) – Huai’an, Jiangsu Province, China, 2021.

Group	CD-RISC score		PTGI score	
	Before intervention	After four weeks of intervention	Before intervention	After four weeks of intervention
Study (n = 102)	52.63 ± 4.34	83.15 ± 2.09^*^	72.16 ± 5.21	98.35 ± 2.56^*^
Control (n = 102)	53.07 ± 4.38	76.54 ± 3.61^*^	72.08 ± 5.17	91.03 ± 4.28^*^
t	0.721	16.004	0.110	14.824
p	0.472	<0.001	0.913	<0.001

*p < 0.05 vs. the same group before intervention.

### QoL and PFD Severity

After four weeks, both groups showed increased WHOQOL-BREF scores and decreased PFDI-20 scores compared to baseline. The study group had higher WHOQOL-BREF scores and lower PFDI-20 scores than the control group (p < 0.05) (see Table 3).

**Table 3:** WHOQOL-BREF and PFDI-20 scores before and after four weeks of intervention (*x̄* ± s, point) – Huai’an, Jiangsu Province, China, 2021.

Group	WHOQOL-BREF score		PFDI-20 score	
	Before intervention	After four weeks of intervention	Before intervention	After four weeks of intervention
Study (n = 102)	69.42 ± 4.17	106.85 ± 7.12^*^	50.75 ± 6.42	35.38 ± 2.63^*^
Control (n = 102)	70.28 ± 4.23	99.47 ± 6.34^*^	51.51 ± 6.27	39.58 ± 4.45^*^
t	1.462	7.818	0.855	8.206
p	0.145	<0.001	0.393	<0.001

*p < 0.05 vs. the same group before intervention.

### Stigma

After four weeks of the intervention, the SIS scores in each dimension and the total score decreased in both groups compared to before the intervention. The scores were lower in the study group than in the control group (p < 0.05) (Table 4).

**Table 4:** SIS score in each dimension and total score before and after four weeks of intervention (*x̄* ± s, point) – Huai’an, Jiangsu Province, China, 2021.

Group		Study (n = 102)	Control (n = 102)	t	p
Internalized shame	Before intervention	15.28 ± 2.13	15.45 ± 2.08	0.577	0.565
	After four weeks of intervention	8.62 ± 1.05*	11.84 ± 1.73*	16.070	<0.001
Social isolation	Before intervention	24.71 ± 1.16	24.88 ± 1.04	1.102	0.272
	After four weeks of intervention	12.39 ± 3.27*	15.95 ± 2.69*	8.491	<0.001
Financial insecurity	Before intervention	8.37 ± 1.12	8.48 ± 1.06	0.720	0.472
	After four weeks of intervention	3.55 ± 0.64*	5.29 ± 0.87*	16.271	<0.001
Social rejection	Before intervention	31.68 ± 1.02	31.52 ± 1.04	1.109	0.269
	After four weeks of intervention	16.59 ± 2.25^*^	20.38 ± 3.53^*^	9.144	<0.001
Total score	Before intervention	79.23 ± 2.06	78.97 ± 2.04	0.906	0.366
	After four weeks of intervention	41.55 ± 3.17^*^	53.29 ± 4.73^*^	20.823	<0.001

*p < 0.05 vs. the same group before intervention.

### Perioperative Indicators and Nursing Satisfaction

The study group had a shorter postoperative exhaustion time, shorter length of stay, and higher nursing satisfaction scores than the control group (p < 0.05) (Table 5).

**Table 5:** Perioperative indicators and nursing satisfaction (*x̄* ± s) – Huai’an, Jiangsu Province, China, 2021.

Group	Postoperative exhaust time (h)	Length of stay (d)	Nursing satisfaction score (point)
Study (n = 102)	20.97 ± 2.25	8.84 ± 0.65	98.81 ± 0.75
Control (n = 102)	31.48 ± 4.61	10.39 ± 1.04	94.46 ± 0.93
t	20.692	12.764	36.772
p	<0.001	<0.001	<0.001

## DISCUSSION

Nursing is a demanding profession that requires excellent clinical skills, good team communication and cooperation, and the ability to adapt to long-term shift work. These stressors can easily lead to burnout, increasing the risk of errors and negatively impacting the quality of care and patient satisfaction^(19)^. Therefore, developing a new nursing management model is clinically significant in alleviating burnout, promoting nursing reforms, and achieving high-quality nursing services.

This study compared the study group with the control group. The study group had shorter postoperative exhaust time and length of stay and higher nursing satisfaction scores. These results suggest that the new nursing organizational management model developed using appreciative inquiry can optimize perioperative indicators and improve nursing satisfaction for gynecological tumor patients undergoing surgery.

The appreciative inquiry approach has been used in various international settings. For example, it has been used to improve support systems in UK nursing homes during the pandemic by involving staff and residents in service co-design^(20)^. Similarly, appreciative inquiry has been used in a pediatric intensive care unit to understand healthcare workers’ perceptions of happiness and well-being, which has ultimately improved care quality^(21)^. These studies support our findings and confirm the effectiveness of appreciative inquiry in managing the healthcare workforce and providing patient care.

This new organizational model reflects a psychological innovation in nursing management. Guided by the 4D cycle, this approach emphasizes positive dialogue, shared vision, and collective strengths^(22)^. During the discovery and dream stages, nurses identify personal and organizational strengths, thereby enhancing their self-efficacy and professional identity^(23)^. The design stage fosters collaboration and innovation, promoting the implementation of high-impact nursing measures. The destiny stage strengthens learning and reflection, cultivating a growth mindset that improves nursing quality and fosters a positive work environment^(24)^.

In this study, the CD-RISC, PTGI, and WHOQOL-BREF scores were higher, while the PFDI-20, SIS, and total SIS scores were lower in the study group than in the control group after four weeks of intervention. This suggests that the new nursing management model constructed using appreciative inquiry can improve PFM function, psychological resilience, and quality of life (QoL) and reduce the stigma experienced by gynecological tumor patients undergoing surgery. Regarding the acute nursing needs and impacts of gynecological tumor patients undergoing radiotherapy, psychosocial risk factors are closely related to an increased frequency of radiotherapy^(25)^. To better address the psychosocial and supportive nursing needs of gynecological tumor patients, targeted strategies should be implemented early on. Similarly, we found that the new nursing management model based on appreciative inquiry can improve the psychological and social functioning of gynecological cancer patients.

These benefits may stem from a multifaceted intervention strategy embedded within the appreciative inquiry framework. First, preoperative activities with themes like “I Know You Know,” “Travel with Trust and Unobstructed Communication,” and “Self and Vision Reconstruction” promoted emotional expression, social support, and surgical confidence. Second, daily imaginative relaxation therapy, which used visual and auditory cues, reduced pain and anxiety while strengthening resilience. Third, continuous nursing care combined with structured aerobic training improved physical function and overall health. Family-involved health education fostered emotional and social support. After the operation, personalized pelvic floor muscle (PFM) rehabilitation combined traditional exercises with urine suppression and biofeedback training to further enhance nerve-muscle recovery and reduce postoperative dysfunction.

Nevertheless, this study has limitations. First, it was conducted in a single medical center, which may limit its generalizability and introduce selection bias. The follow-up period was short and focused only on early postoperative outcomes, so the long-term effects remain unclear. Furthermore, subjective scales used to measure satisfaction and quality of life may be subject to reporting bias. Future multicenter studies with longer follow-up periods are needed to validate these results.

## CONCLUSION

In conclusion, the new nursing management model developed using appreciative inquiry is high-quality with significant clinical application value. This model can optimize perioperative indicators, improve patient-family decision-making (PFD), enhance psychological resilience and posttraumatic growth levels, improve quality of life (QoL), reduce stigma, and promote overall nursing satisfaction among gynecological tumor patients undergoing surgery.

## Data Availability

All data are available upon reasonable request to the corresponding author.
