# Supplementary material for: Effect of a new nursing organization management mode constructed by appreciative inquiry on gynecological tumor patients in the perioperative period
Source: Rev Esc Enferm USP. 2025 Oct 27;59:e20250016. doi: 10.1590/1980-220X-REEUSP-2025-0016en (PMC12571229; doi:10.1590/1980-220X-REEUSP-2025-0016en)
Supplement: Translation

# Translation: Efecto de un nuevo modelo de gestión de la organización de enfermería basado en la investigación apreciativa en pacientes con tumores ginecológicos en el periodo perioperatório

**DOI:** 10.1590/1980-220X-REEUSP-2025-0016es

**Authors:** Chuntao Fang, Jing Yang

**Affiliations:** 1 Huai’an Second People’s Hospital, Department of Gynecology, Huai’an, China.

**Keywords:** Encuestas y Cuestionarios, Ginecología, Neoplasias, Enfermería, Gestión en Salud, Periodo Perioperatorio

## Abstract

**Objetivo:**

Evaluar el efecto de un nuevo modelo de gestión de la organización de enfermería basado en la investigación apreciativa en pacientes con tumores ginecológicos durante el periodo perioperatorio.

**Método:**

Se realizó un análisis retrospectivo de 204 pacientes con tumores ginecológicos que se sometieron a tratamiento quirúrgico entre junio de 2021 y junio de 2024. Las pacientes fueron asignadas a un grupo de control (n = 102; modelo de gestión de la organización de enfermería rutinario) o a un grupo de estudio (n = 102; nuevo modelo de gestión de la organización de enfermería construido mediante investigación apreciativa) según su modelo de enfermería durante el periodo perioperatorio.

**Resultados:**

Ambos grupos obtuvieron puntuaciones más altas en la Escala de Calidad de Vida de la Organización Mundial de la Salud (WHOQOL-BREF) y puntuaciones más bajas en el Inventario de Malestar Pélvico-Formulario Corto 20 (PFDI-20) en comparación con antes de la intervención. El grupo de estudio obtuvo puntuaciones más altas en la WHOQOL-BREF y puntuaciones más bajas en el PFDI-20 que el grupo de control (p < 0,05). Las puntuaciones en la Escala de Impacto Social en cada dimensión y la puntuación total disminuyeron en ambos grupos en comparación con el valor inicial, observándose puntuaciones más bajas en el grupo de estudio que en el grupo de control (p < 0,05).

**Conclusión:**

El nuevo modelo de gestión organizativa de enfermería desarrollado mediante la investigación apreciativa puede optimizar los indicadores perioperatorios, mejorar la disfunción del suelo pélvico y aumentar la resiliencia psicológica.

## INTRODUCCIÓN

El cáncer de ovario (CO), el cáncer de cuello uterino (CCU), el cáncer de endometrio (CE) y los fibromas uterinos (FU) son tumores comunes del sistema reproductivo femenino. Las pacientes suelen presentar compresión pélvica, sangrado vaginal y, en casos graves, disfunción del suelo pélvico (DSP), lo que tiene un grave impacto adverso en las funciones psicológicas, fisiológicas y sociales y en la calidad de vida (CV) de las pacientes^(1,2)^. Actualmente, la cirugía es un tratamiento importante para los tumores ginecológicos. Sin embargo, dado que el útero y los ovarios son órganos vitales para las mujeres, y que la cirugía es invasiva, la mayoría de las pacientes pueden experimentar miedo, ansiedad y otras emociones negativas debido a la preocupación por el trauma quirúrgico, la recuperación posoperatoria y la fertilidad. Esto aumenta el riesgo de respuestas de estrés cardiovascular intraoperatorio y dificulta el éxito de la cirugía y la recuperación posoperatoria^(3,4)^. Por lo tanto, las medidas de enfermería activas y eficaces tomadas durante el periodo perioperatorio son muy importantes para estabilizar las emociones de las pacientes y ayudarlas a desarrollar estrategias de afrontamiento positivas.

La investigación apreciativa es un enfoque eficaz para promover el cambio organizativo. Lo hace descubriendo y activando sistemáticamente la máxima eficiencia y capacidad de la vida organizativa^(5, 6)^. La investigación apreciativa desempeña un papel importante en la mejora de la gestión de los recursos humanos, el fortalecimiento de la capacidad de los equipos, el desarrollo de la práctica clínica de enfermería y la promoción del cambio organizativo en la enfermería. En un estudio cualitativo realizado con internos de la unidad de cuidados intensivos durante la pandemia, la investigación apreciativa llevó a los internos a considerar y rediseñar activamente su flujo de trabajo, el apoyo laboral y el desarrollo personal, lo que en última instancia les ayudó a alcanzar los objetivos organizativos^(7)^. El concepto de investigación apreciativa también se ha aplicado al cambio organizacional en la atención primaria de salud bucodental, utilizando un modelo de ciclo 4D (Discovery-Dream-Design-Destiny; descubrimiento, sueño, diseño, destino) a lo largo de todo el proceso^(8)^. Al mejorar la gestión organizacional y aumentar los recursos, se reforzó el deseo de los miembros de la organización de integrar la salud bucodental en la atención primaria, lo que permitió implementar con éxito el plan estratégico organizacional. La investigación apreciativa se ha introducido para ayudar a los médicos de familia canadienses a proporcionar el mejor apoyo a los pacientes que necesitan cuidados paliativos en los centros médicos comunitarios^(9)^. La investigación apreciativa resultó más eficaz que otros métodos de gestión para fortalecer la comunicación y fomentar el trabajo en equipo entre los cuidadores, como los médicos de familia, y para ayudar a establecer buenas relaciones entre el personal de enfermería y los pacientes, mejorando así la calidad de los cuidados paliativos.

En este estudio, se aplicó un nuevo modelo de gestión organizativa de enfermería basado en la investigación apreciativa a la intervención de enfermería para pacientes con tumores ginecológicos sometidas a cirugía, y se evaluaron sus efectos en los indicadores perioperatorios, el estado psicológico, la calidad de vida (CV) y la satisfacción de la enfermería.

## MÉTODO

### Diseño del Estudio y Sujetos

En este estudio retrospectivo, el tamaño de la muestra requerido se determinó basándose en la ecuación para comparar dos proporciones: n = 2 × (Z_α/2_ + Z_β_)^2^ × p(1 – p)/d^2^ donde n = tamaño de la muestra por grupo, Z_α/2_ = valor Z correspondiente al nivel de significación deseado (para α = 0,05, bilateral, Z_α/2_ = 1,96), Z_β_ = valor Z correspondiente a la potencia (para potencia = 80%, Z_β_ = 0,84), P es la tasa de incidencia estimada en el grupo de control, basada en estudios previos o datos piloto, y se supone que es del 50%. D es la diferencia clínicamente significativa prevista entre los grupos, establecida en una mejora del 20%. Utilizando esta ecuación, se calculó que n era 98. Teniendo en cuenta una tasa de abandono prevista de aproximadamente el 5%, el tamaño final de la muestra se ajustó a 102 participantes por grupo, con un total de 204 participantes.

Entre junio de 2021 y junio de 2024, se recolectaron datos de 204 pacientes con tumores ginecológicos sometidas a tratamiento quirúrgico en nuestro hospital. Según los diferentes modos de enfermería en el periodo perioperatorio, las pacientes fueron asignadas a un grupo de control (n = 102; modo de gestión de la organización de enfermería rutinario) o a un grupo de estudio (n = 102; nuevo modo de gestión de la organización de enfermería construido mediante investigación apreciativa).

### Subsección de Aspectos éticos

El Comité de Ética en Investigación del Segundo Hospital Popular de Huai’an aprobó este estudio (n.º JSHASH2021105).

### Criterios de Inclusión y Exclusión

Los criterios de inclusión fueron los siguientes: 1) Pacientes que cumplían los criterios diagnósticos de CO, CCU, CE o FU^(10, 11, 12, 13)^ y que fueron confirmados por:

- Histeroscopia (26105FA, KARL STORZ, Alemania)
- Instrumento de diagnóstico por ultrasonido Doppler color (IU22, Philips, Países Bajos)
- Colposcopia (DKY-1611, Dalian Care Medical Equipment Co., Ltd.) y biopsia patológica; 2) pacientes con datos clínicos completos; 3) pacientes sometidos a tratamiento quirúrgico por primera vez; y 4) pacientes sin trastornos psiquiátricos (por ejemplo, esquizofrenia o trastorno bipolar).

Los criterios de exclusión fueron: 1) pacientes incapaces de cooperar en la realización de la escala debido a trastornos cognitivos o de comunicación; 2) pacientes que desarrollaron otras enfermedades graves durante la intervención; 3) pacientes con complicaciones derivadas de otras enfermedades neoplásicas; 4) pacientes con una esperanza de vida estimada inferior a seis meses; 5) pacientes con complicaciones derivadas de metástasis en órganos distantes (por ejemplo, pulmón, hígado, cerebro); y 6) pacientes menores de 20 años), 7) disfunción grave del hígado, los riñones, el corazón o los pulmones, 8) deterioro grave de la función de las extremidades u otras enfermedades que afectan gravemente al movimiento de las extremidades (por ejemplo, osteoartritis), 9) edad <20 años, y 10) enfermedades hematológicas, transmisibles o infecciosas.

### Modo de Manejo para el Grupo de Control

Se adoptó un modelo de manejo organizativo de enfermería rutinario. En concreto, se distribuyeron a las pacientes, en el momento de su ingreso, folletos de salud en los que se detallaban las causas, los riesgos, los protocolos quirúrgicos y las precauciones posoperatorias de los tumores ginecológicos. Los folletos también instruían a las pacientes para que se relajaran y aceptaran la cirugía. Entre siete y diez días después de la cirugía, se guió y ayudó a las pacientes en el entrenamiento rutinario de rehabilitación de los músculos del suelo pélvico. Esto consistía en contraer conscientemente los músculos vaginales, uretrales y anales después de vaciar la vejiga durante cinco a diez segundos, seguido de diez segundos de relajación. Las pacientes realizaban este ejercicio entre diez y quince minutos por sesión, tres veces al día. La intervención duró cuatro semanas.

### Modo de Gestión Para el Grupo de Estudio

Adoptamos un nuevo modo de gestión de la organización de enfermería construido mediante la investigación apreciativa. Nuestra institución introdujo este modelo a través de un proceso de implementación estructurado. En primer lugar, los directores de enfermería y el personal básico recibieron una formación específica. A continuación, se formó un equipo dedicado a la mejora de la calidad de la enfermería. El equipo dirigió entonces talleres y debates en todo el departamento para identificar los puntos fuertes, establecer objetivos comunes y rediseñar los procesos de enfermería perioperatoria de forma colaborativa. Se desarrollaron protocolos estandarizados y modelos de comunicación basados en los principios de la investigación apreciativa, que se incorporaron a la práctica diaria.

1) Descubrimiento: El personal médico experimentado participó en una formación profesional sobre el método de la investigación apreciativa para comprender el ciclo 4D (descubrimiento, sueño, diseño, destino). Se creó un ambiente de reunión relajado y armonioso, y se animó a todo el personal médico de forma positiva y optimista a hablar de casos impresionantes y de sus efectos positivos en su trabajo, compartiendo experiencias de enfermería de alta calidad.

2) Sueño: Basándose en el intercambio de las mejores experiencias de enfermería, se animó al personal de enfermería a imaginar cómo sería el trabajo de enfermería en los próximos cinco a diez años, combinando sus propias ventajas y potencial. También se les animó a rediseñar con audacia la asignación de funciones personales, la estructura organizativa del departamento y los procesos de trabajo de enfermería. Los miembros del equipo debatieron formas de alcanzar los objetivos de la organización y se ayudaron mutuamente a convertir sus ideas en medidas específicas y orientadas a los resultados.

3) Diseño: A través de entrevistas individuales en profundidad, combinadas con las mejores condiciones de trabajo, el potencial personal y las ventajas organizativas, se animó al personal de enfermería a considerar cómo promover la realización de la visión futura. Los miembros del equipo formularon conjuntamente una serie de protocolos de enfermería eficaces y viables, concretamente: a) Formación en Músculo del Suelo Pélvico (pelvic floor muscle PFM): Basándose en la formación rutinaria en PFM, se impartió formación en supresión de la orina y biofeedback a partir del quinto día después de la operación, según el estado de los pacientes. Estas sesiones de entrenamiento contaron con la ayuda del instrumento eléctrico de biofeedback PFM (Huayu Medical Technology [Wuhan] Co., Ltd., HD-V8, EHXB 20190157). Se explicaron detalladamente el contenido del entrenamiento, los métodos de operación específicos y las precauciones pertinentes, teniendo en cuenta el nivel educativo de las pacientes. b) Enfermería continua: se crearon grupos de WeChat entre enfermeras y pacientes, así como cuentas oficiales de WeChat para enviar regularmente mensajes sobre precauciones posoperatorias, vídeos de entrenamiento de rehabilitación y medidas de enfermería posoperatoria para tumores ginecológicos. Se animó a los familiares a cuidar y supervisar a los pacientes en su entrenamiento de rehabilitación. Se impartieron regularmente conferencias especiales sobre ejercicio, a las que se invitó a asistir a los familiares. Durante estas conferencias, se introdujeron entrenamientos de rehabilitación como subir y bajar escaleras, ejercicios de musculación y respiración abdominal. Se invitó a pacientes con una buena recuperación posoperatoria a compartir sus experiencias. c) Enfermería psicológica: Se llevaron a cabo actividades de educación sanitaria con el tema “Yo sé que tú sabes” tres días antes de la operación. Se animó a los pacientes a presentarse y comunicarse entre sí. Se explicó a los pacientes con tumores ginecológicos la relación entre la rehabilitación posoperatoria y el manejo emocional mediante videos y presentaciones de PowerPoint. Se llevó a cabo una segunda actividad, “Viajar con confianza y comunicación sin obstáculos”, dos días antes de la cirugía para animar a los pacientes a comunicarse, experimentar diferentes modos de comunicación y promover un flujo de información positivo. Se celebró una reunión de intercambio entre pacientes con el tema “Reconstrucción del yo y la visión” un día antes de la cirugía para animar a los pacientes a imaginar una vida mejor después de la cirugía y compartir sus ideas con los demás. d) Entrenamiento de relajación imaginativa: se guió a las pacientes a través de un entrenamiento de relajación utilizando imágenes del mar, la playa y otras escenas, así como música de fondo. Por ejemplo, se pidió a las pacientes que imaginaran una escena costera con imágenes de puestas de sol y cocoteros, mientras se reproducía de fondo música con sonidos de olas del mar y cocoteros meciéndose. También se proporcionaron palabras de orientación: “Estás caminando por la orilla del mar con buenos amigos, disfrutando de la puesta de sol y las nubes. Sopla la brisa y todo es hermoso...”. Esto se hacía una vez antes de acostarse por la noche o antes de la siesta de la tarde.

4) Destino: Se compartieron las mejores experiencias y logros de enfermería para ayudar al personal de enfermería a generar esperanza en torno a objetivos de enfermería profundos y visiones profesionales. Esto les ayudó a mantener una motivación positiva para el aprendizaje y el ajuste continuos, así como a participar activamente en ejercicios de escenas entre enfermeras y pacientes para mejorar las habilidades de comunicación. La intervención duró cuatro semanas.

Para garantizar la coherencia en la aplicación del modelo de gestión de la organización de enfermería basado en la investigación apreciativa durante el periodo de estudio de tres años, se elaboró y aplicó un manual de formación y un protocolo de implementación estandarizados. Todas las enfermeras recién contratadas del grupo de estudio recibieron una formación inicial estructurada que incluía la teoría de la investigación apreciativa, el proceso del ciclo 4D y aplicaciones prácticas adaptadas a la atención perioperatoria. Además, se llevaron a cabo reuniones mensuales de revisión de la calidad y sesiones de formación de actualización para mantener la fidelidad al enfoque de la investigación apreciativa y garantizar que se respetaran los principios básicos a pesar de la rotación del personal.

### Evaluación de los Resultados

1. Se evaluaron los siguientes indicadores antes y después de la intervención de cuatro semanas:
  - Resiliencia psicológica: La resiliencia psicológica se evaluó utilizando la Escala de Resiliencia de Connor-Davidson (Connor-Davidson Resilience Scale – CD-RISC) de 25 ítems, que abarca cinco dimensiones: competencia personal/tenacidad (ocho ítems)^(14)^, aceptación positiva del cambio (cinco ítems), tolerancia al afecto negativo (siete ítems), influencias espirituales (dos ítems) y autocontrol (tres ítems). Los ítems se puntúan en una escala Likert de 5 puntos: nunca (0), rara vez (1), a veces (2), a menudo (3) y siempre (4). Las puntuaciones posibles oscilan entre 0 y 100, y las puntuaciones más altas indican una mayor resiliencia.
  - Nivel de crecimiento postraumático: Las actitudes de pensamiento positivo postraumático se evaluaron utilizando el Inventario de Crecimiento Postraumático (Posttraumatic Growth Inventory PTGI) de 21 ítems, que consta de cinco dimensiones^(15)^: cambio espiritual (dos ítems), relación con los demás (siete ítems), nuevas posibilidades (cinco ítems), apreciación de la vida (tres ítems) y fortaleza personal (cuatro ítems). Los ítems se puntúan en una escala Likert de seis puntos que va de 0 (nunca) a 5 (máximo). Las puntuaciones oscilan entre 21 y 126, y las puntuaciones más altas indican un cambio psicológico positivo y un crecimiento más fuertes.
  - Calidad de vida: La calidad de vida de los dos grupos se evaluó utilizando la Versión Breve de la Escala de Calidad de Vida de la Organización Mundial de la Salud (World Health Organization Quality of Life Scale Brief Version – WHOQOL-BREF), que incluye 26 ítems en cuatro dimensiones: relaciones sociales (tres ítems)^(16)^, entorno (ocho ítems), psicología (seis ítems) y salud física (siete ítems). Los ítems se puntúan en una escala Likert de 5 puntos (1–5 puntos), y algunos ítems se puntúan a la inversa. Las puntuaciones oscilan entre 26 y 130 puntos; una puntuación más baja indica una peor calidad de vida.
  - Gravedad del malestar del suelo pélvico (Pelvic Floor Distress PFD): Se utilizó el Inventario de malestar del suelo pélvico-Formulario corto 20 (PFDI-20) para evaluar la gravedad del PFD en los dos grupos^(17)^. La escala consta de 20 ítems, cada uno de los cuales se puntúa en una escala Likert de 4 puntos: sin impacto (1), impacto leve (2), impacto moderado (3) e impacto grave (4). Las puntuaciones oscilan entre 20 y 80, y las puntuaciones más bajas indican un PFD más leve.
  - Estigma: El estigma experimentado por los dos grupos se evaluó utilizando la Escala de Impacto Social (Social Impact Scale – SIS) de 24 ítems, que incluye cuatro dimensiones^(18)^: vergüenza internalizada (cinco ítems, 5–20 puntos); aislamiento social (siete ítems, 7–28 puntos); inseguridad financiera (tres ítems, 3–12 puntos); y rechazo social (nueve ítems, 9–36 puntos). Cada ítem se puntúa en una escala de cuatro puntos, con una puntuación total que oscila entre 24 y 96 puntos. Una puntuación más alta indica un mayor estigma.
2. Indicadores perioperatorios y satisfacción con la enfermería: Se registraron el tiempo de agotamiento posoperatorio y la duración de la estadía de los dos grupos. Se utilizó un cuestionario diseñado por el hospital para evaluar la satisfacción con la enfermería, que abarcaba cuatro dimensiones: actitud de la enfermería, nivel de enfermería, educación sanitaria y gestión de la enfermería. La puntuación total posible era de 100 puntos, y las puntuaciones más altas indicaban una mayor satisfacción. Para garantizar la calidad del cuestionario, se llevó a cabo una evaluación preliminar de su fiabilidad y validez. La fiabilidad de la consistencia interna se evaluó utilizando el alfa de Cronbach, que resultó ser de 0,86, lo que indica una buena fiabilidad. La validez del contenido fue evaluada por un panel de cinco expertos en enfermería, lo que dio como resultado un índice de validez del contenido de 0,89. Estos resultados respaldaron la fiabilidad y validez del cuestionario para su uso en este estudio.

### Análisis Estadístico

Para el análisis se utilizó el software SPSS 23.0. Los datos de medición se describieron utilizando la media ± desviación estándar (x ± s) y se compararon utilizando una prueba t para muestras independientes entre dos grupos y una prueba t para muestras emparejadas dentro del grupo. Los datos de recuento se describieron como un porcentaje [n (%)] y se sometieron a una prueba de chi cuadrado. No se realizaron análisis multivariantes ni análisis de subgrupos/interacción, lo que puede limitar el control de posibles factores de confusión. Los datos faltantes fueron mínimos (menos del 5%) y se manejaron mediante un análisis de casos completos. Se consideró estadísticamente significativo un valor p bilateral <0,05.

## RESULTADOS

### Datos Clínicos Basales

No hubo diferencias significativas en el nivel educativo, la edad, el curso de la enfermedad, el tipo de tumor, el índice de masa corporal, el método quirúrgico o el estado civil entre los dos grupos (p > 0,05) (Tabla 1).

**Tabla 1:** Datos generales de dos grupos – Huai’an, provincia de Jiangsu, China, 2021.

Indicador		Grupo en estudio (n = 102)	Grupo control (n = 102)	Valor estadístico	valor de p
Nivel educativo [n (%)]	Segundo ciclo y superior	58 (56.86)	53 (51.96)	χ^2^ = 0.494	0.482
	Primer ciclo o menos	44 (43.14)	49 (48.04)		
Edad (x̄ ± s, años)		52.57 ± 6.13	53.04 ± 6.18	t = 0.545	0.586
Tiempo de enfermedad (x̄ ± s, mes)		10.66 ± 0.98	10.74 ± 1.05	t = 0.563	0.574
Tipo de tumor [n (%)]	CO	7 (6.86)	6 (5.88)	χ^2^ = 1.203	0.752
	CCU	8 (7.84)	7 (6.86)		
	CE	10 (9.80)	15 (14.71)		
	FU	77 (75.49)	74 (72.55)		
Índice de masa corporal (x̄ ± s, kg/m^2^)		22.14 ± 0.85	22.25 ± 0.88	t = 0.908	0.365
Tipo de cirugía [n (%)]	Cirugía radical	51 (50.00)	46 (45.10)	χ^2^ = 0.498	0.780
	Cirugía de preservación del útero	25 (24.51)	27 (26.47)		
	Histerectomía total	26 (25.49)	29 (28.43)		
Estado civil [n (%)]	Casada	87 (85.29)	83 (81.37)	c^2^ = 1.272	0.529
	Soltera	9 (8.82)	14 (13.73)	c^2^ = 0.498	0.780
	Divorciada/viuda/separada	51 (50.00)	46 (45.10)		

### Resiliencia Psicológica y Nivel de Crecimiento Postraumático

Tras cuatro semanas de intervención, ambos grupos obtuvieron puntuaciones más altas en el CD-RISC y el PTGI que antes de la intervención. El grupo en estudio obtuvo puntuaciones más altas que el grupo de control (p < 0,05) (Tabla 2).

**Tabla 2:** Puntuaciones en el CD-RISC y el PTGI antes y después de cuatro semanas de intervención (*x̄* ± s, punto) – Huai’an, provincia de Jiangsu, China, 2021.

Grupo	puntuaciones CD-RISC		puntuaciones PTGI	
	Antes de intervención	Después de cuatro semanas de intervención	Antes de intervención	Después de cuatro semanas de intervención
Estudio (n = 102)	52.63 ± 4.34	83.15 ± 2.09^*^	72.16 ± 5.21	98.35 ± 2.56^*^
Control (n = 102)	53.07 ± 4.38	76.54 ± 3.61^*^	72.08 ± 5.17	91.03 ± 4.28^*^
t	0.721	16.004	0.110	14.824
p	0.472	<0.001	0.913	<0.001

*p < 0,05 vs. el mismo grupo antes de la intervención.

### Calidad de Vida y Gravedad de la PFD

Después de cuatro semanas, ambos grupos mostraron un aumento en las puntuaciones del WHOQOL-BREF y una disminución en las puntuaciones del PFDI-20 en comparación con los valores iniciales. El grupo de estudio obtuvo puntuaciones más altas en el WHOQOL-BREF y puntuaciones más bajas en el PFDI-20 que el grupo de control (p < 0,05) (véase la tabla 3).

**Tabla 3:** Puntuaciones en el WHOQOL-BREF y el PFDI-20 antes y después de cuatro semanas de intervención (*x̄* ± s, punto) – Huai’an, provincia de Jiangsu, China, 2021.

Grupo	puntuación WHOQOL-BREF		puntuación PFDI-20	
	Antes de intervención	Después de cuatro semanas de intervención	Antes de intervención	Después de cuatro semanas de intervención
Estudio (n = 102)	69.42 ± 4.17	106.85 ± 7.12^*^	50.75 ± 6.42	35.38 ± 2.63^*^
Control (n = 102)	70.28 ± 4.23	99.47 ± 6.34^*^	51.51 ± 6.27	39.58 ± 4.45^*^
t	1.462	7.818	0.855	8.206
p	0.145	<0.001	0.393	<0.001

*p < 0,05 vs. el mismo grupo antes de la intervención.

### Estigma

Tras cuatro semanas de intervención, las puntuaciones SIS en cada dimensión y la puntuación total disminuyeron en ambos grupos en comparación con antes de la intervención. Las puntuaciones fueron más bajas en el grupo de estudio que en el grupo de control (p < 0,05) (Tabla 4).

**Tabla 4:** Puntuación SIS en cada dimensión y puntuación total antes y después de cuatro semanas de intervención (*x̄* ± s, punto) – Huai’an, provincia de Jiangsu, China, 2021.

Grupo		Estudio (n = 102)	Control (n = 102)	t	p
Vergüenza internalizada	Antes de intervención	15.28 ± 2.13	15.45 ± 2.08	0.577	0.565
	Después de cuatro semanas de intervención	8.62 ± 1.05^*^	11.84 ± 1.73^*^	16.070	<0.001
Aislamiento social	Antes de intervención	24.71 ± 1.16	24.88 ± 1.04	1.102	0.272
	Después de cuatro semanas de intervención	12.39 ± 3.27^*^	15.95 ± 2.69^*^	8.491	<0.001
Inseguridad financiera	Antes de intervención	8.37 ± 1.12	8.48 ± 1.06	0.720	0.472
	Después de cuatro semanas de intervención	3.55 ± 0.64^*^	5.29 ± 0.87^*^	16.271	<0.001
Rechazo social	Antes de intervención	31.68 ± 1.02	31.52 ± 1.04	1.109	0.269
	Después de cuatro semanas de intervención	16.59 ± 2.25^*^	20.38 ± 3.53^*^	9.144	<0.001
Puntuación total	Antes de intervención	79.23 ± 2.06	78.97 ± 2.04	0.906	0.366
	Después de cuatro semanas de intervención	41.55 ± 3.17^*^	53.29 ± 4.73^*^	20.823	<0.001

*p < 0,05 vs, el mismo grupo antes de la intervención.

### Indicadores Perioperatorios y Satisfacción de la Enfermería

El grupo de estudio presentó un tiempo de agotamiento posoperatorio más corto, una duración de la estadía más breve y puntuaciones de satisfacción de la enfermería más altas que el grupo de control (P<0,05) (Tabla 5).

**Tabla 5:** Indicadores perioperatorios y satisfacción de la enfermería (*x̄* ± s) – Huai’an, provincia de Jiangsu, China, 2021.

Grupo	Tiempo de agotamiento post-operatorio. (horas)	Duración de la estadía (días)	Puntuación de satisfacción de enfermería (puntos)
Estudio (n = 102)	20.97 ± 2.25	8.84 ± 0.65	98.81 ± 0.75
Control (n = 102)	31.48 ± 4.61	10.39 ± 1.04	94.46 ± 0.93
t	20.692	12.764	36.772
p	<0.001	<0.001	<0.001

## DISCUSIÓN

La enfermería es una profesión exigente que requiere excelentes habilidades clínicas, buena comunicación y cooperación en equipo, y la capacidad de adaptarse a turnos de trabajo prolongados. Estos factores estresantes pueden conducir fácilmente al agotamiento, lo que aumenta el riesgo de errores y afecta negativamente a la calidad de la atención y a la satisfacción de los pacientes^(19)^. Por lo tanto, el desarrollo de un nuevo modelo de gestión de enfermería es clínicamente significativo para aliviar el agotamiento, promover reformas en la enfermería y lograr servicios de enfermería de alta calidad.

Este estudio comparó el grupo de estudio con el grupo de control. El grupo de estudio tuvo un tiempo de agotamiento postoperatorio y una duración de la estadía más cortos, así como puntuaciones más altas en satisfacción con la enfermería. Estos resultados sugieren que el nuevo modelo de gestión organizativa de la enfermería desarrollado mediante la investigación apreciativa puede optimizar los indicadores perioperatorios y mejorar la satisfacción con la enfermería de las pacientes con tumores ginecológicos sometidas a cirugía.

El enfoque de la investigación apreciativa se ha utilizado en diversos entornos internacionales. Por ejemplo, se ha utilizado para mejorar los sistemas de apoyo en las residencias de ancianos del Reino Unido durante la pandemia, involucrando al personal y a los residentes en el diseño conjunto de los servicios^(20)^. Del mismo modo, la investigación apreciativa se ha utilizado en una unidad de cuidados intensivos pediátricos para comprender las percepciones de felicidad y bienestar de los trabajadores sanitarios, lo que en última instancia ha mejorado la calidad de la atención^(21)^. Estos estudios respaldan nuestros hallazgos y confirman la eficacia de la investigación apreciativa en la gestión del personal sanitario y la prestación de atención al paciente.

Este nuevo modelo organizativo refleja una innovación psicológica en la gestión de la enfermería. Guiado por el ciclo 4D, este enfoque hace hincapié en el diálogo positivo, la visión compartida y las fortalezas colectivas^(22)^. Durante las etapas de descubrimiento y sueño, las enfermeras identifican las fortalezas personales y organizativas, lo que mejora su autoeficacia e identidad profesional^(23)^. La etapa de diseño fomenta la colaboración y la innovación, promoviendo la implementación de medidas de enfermería de alto impacto. La etapa del destino refuerza el aprendizaje y la reflexión, cultivando una mentalidad de crecimiento que mejora la calidad de la enfermería y fomenta un entorno de trabajo positivo^(24)^.

En este estudio, las puntuaciones del CD-RISC, el PTGI y el WHOQOL-BREF fueron más altas, mientras que las puntuaciones del PFDI-20, el SIS y el SIS total fueron más bajas en el grupo de estudio que en el grupo de control después de cuatro semanas de intervención. Esto sugiere que el nuevo modelo de gestión de enfermería construido utilizando la investigación apreciativa puede mejorar la función del músculo del piso pélvico, la resiliencia psicológica y la calidad de vida (CV) y reducir el estigma que experimentan las pacientes con tumores ginecológicos sometidas a cirugía. En cuanto a las necesidades de enfermería agudas y los impactos de las pacientes con tumores ginecológicos sometidas a radioterapia, los factores de riesgo psicosociales están estrechamente relacionados con una mayor frecuencia de radioterapia^(25)^. Para abordar mejor las necesidades psicosociales y de apoyo de las pacientes con tumores ginecológicos, se deben implementar estrategias específicas desde el principio. Del mismo modo, hemos descubierto que el nuevo modelo de gestión de enfermería basado en la investigación apreciativa puede mejorar el funcionamiento psicológico y social de las pacientes con cáncer ginecológico.

Estos beneficios pueden derivarse de una estrategia de intervención multifacética integrada en el marco de la investigación apreciativa. En primer lugar, las actividades preoperatorias con temas como “Sé que tú sabes”, “Viaja con confianza y comunicación sin obstáculos” y “Reconstrucción del yo y la visión” promovieron la expresión emocional, el apoyo social y la confianza en la cirugía. En segundo lugar, la terapia de relajación imaginativa diaria, que utilizaba señales visuales y auditivas, redujo el dolor y la ansiedad al tiempo que reforzó la resiliencia. En tercer lugar, la atención de enfermería continua, combinada con un entrenamiento aeróbico estructurado, mejoró la función física y la salud general. La educación sanitaria en la que participó la familia fomentó el apoyo emocional y social. Después de la operación, la rehabilitación personalizada de los músculos del suelo pélvico (PFM) combinó ejercicios tradicionales con supresión de la orina y entrenamiento de biofeedback para mejorar aún más la recuperación nerviosa y muscular y reducir la disfunción posoperatoria.

No obstante, este estudio tiene limitaciones. En primer lugar, se llevó a cabo en un único centro médico, lo que puede limitar su generalización e introducir un sesgo de selección. El periodo de seguimiento fue breve y se centró únicamente en los resultados postoperatorios tempranos, por lo que los efectos a largo plazo siguen sin estar claros. Además, las escalas subjetivas utilizadas para medir la satisfacción y la calidad de vida pueden estar sujetas a un sesgo de notificación. Se necesitan futuros estudios multicéntricos con periodos de seguimiento más largos para validar estos resultados.

## CONCLUSIÓN

En conclusión, el nuevo modelo de gestión de enfermería desarrollado mediante la investigación apreciativa es de alta calidad y tiene un importante valor de aplicación clínica. Este modelo puede optimizar los indicadores perioperatorios, mejorar la toma de decisiones del paciente y la familia, aumentar la resiliencia psicológica y los niveles de crecimiento postraumático, mejorar la calidad de vida, reducir el estigma y promover la satisfacción general con la enfermería entre las pacientes con tumores ginecológicos sometidas a cirugía.

## Data Availability

Todos los datos están disponibles previa solicitud razonable al autor correspondiente.
